# Association between environmental factors and dengue incidence in Lao People’s Democratic Republic: a nationwide time-series study

**DOI:** 10.1186/s12889-023-17277-0

**Published:** 2023-11-27

**Authors:** Masumi Sugeno, Erin C. Kawazu, Hyun Kim, Virasack Banouvong, Nazife Pehlivan, Daniel Gilfillan, Ho Kim, Yoonhee Kim

**Affiliations:** 1 Department of Global Environmental Health, Graduate School of Medicine, The University of Tokyo, 7-3-1 Hongo, Bunkyo-Ku, Tokyo, 113-0033 Japan; 2 Institute for Global Environmental Strategies, Hayama, Japan; 3 School of Public Health, University of Minnesota Twin Cities, Minneapolis, USA; 4 Lao PDR Centre for Malariology, Parasitology and Entomology, Vientiane Capital, Lao People’s Democratic Republic; 5 Graduate School of Public Health, Seoul National University, 1 Gwanak-Ro, Gwanak-Gu, Seoul, 151-742 South Korea; 6 Fenner School of Environment and Society, Australian National University, Australian Capital Territory, Canberra, Australia

**Keywords:** Temperature, Rainfall, Risk assessment, Vector-borne diseases, Dengue

## Abstract

**Background:**

Dengue fever is a vector-borne disease of global public health concern, with an increasing number of cases and a widening area of endemicity in recent years. Meteorological factors influence dengue transmission. This study aimed to estimate the association between meteorological factors (i.e., temperature and rainfall) and dengue incidence and the effect of altitude on this association in the Lao People’s Democratic Republic (Lao PDR).

**Methods:**

We used weekly dengue incidence and meteorological data, including temperature and rainfall, from 18 jurisdictions in Lao PDR from 2015 to 2019. A two-stage distributed lag nonlinear model with a quasi-Poisson distribution was used to account for the nonlinear and delayed associations between dengue incidence and meteorological variables, adjusting for long-term time trends and autocorrelation.

**Results:**

A total of 55,561 cases were reported in Lao PDR from 2015 to 2019. The cumulative relative risk for the 90^th^ percentile of weekly mean temperature (29 °C) over 22 weeks was estimated at 4.21 (95% confidence interval: 2.00–8.84), relative to the 25^th^ percentile (24 °C). The cumulative relative risk for the weekly total rainfall over 12 weeks peaked at 82 mm (relative risk = 1.76, 95% confidence interval: 0.91–3.40) relative to no rain. However, the risk decreased significantly when heavy rain exceeded 200 mm. We found no evidence that altitude modified these associations.

**Conclusions:**

We found a lagged nonlinear relationship between meteorological factors and dengue incidence in Lao PDR. These findings can be used to develop climate-based early warning systems and provide insights for improving vector control in the country.

**Supplementary Information:**

The online version contains supplementary material available at 10.1186/s12889-023-17277-0.

## Introduction

Dengue is a vector-borne disease of global public health concern, with an increasing number of cases and a widening area of endemicity in recent years. It is a viral infection transmitted to humans through the bites of infected mosquitoes that has spread across tropical and subtropical regions over the past 60 years, threatening over half of the world’s population [1, 2]. The primary vectors are the *Aedes aegypti* and, to a lesser extent, the *Aedes albopictus* [3].

The Lao People’s Democratic Republic (Lao PDR) is among the top four countries in Southeast Asia with the highest age-standardized incidence of dengue [4]. Southeast Asian countries, including Lao PDR, are prone to dengue epidemics, and endemic areas are burdened by increased healthcare costs and reduced productivity [5]. Because the disease burden of dengue is particularly severe in countries and regions with limited resources, understanding the spatiotemporal patterns of dengue incidence and identifying the key determinants of disease outbreaks would be helpful for appropriate resource allocation in vulnerable areas.

While various factors affect dengue transmission, meteorological indicators are important, as they affect mosquito breeding rates and virus replication rates inside mosquitoes [6]. For example, temperature and rainfall determine the optimal habitat conditions for mosquitoes [7, 8]. In addition, temperature and rainfall are reported to be associated with dengue incidence in tropical regions, including Southeast Asia. Several studies have reported that high temperatures increase the risk of dengue infection, and that extreme rainfall decreases the risk of dengue. However, the association between these meteorological factors and dengue was found to vary across region [9, 10]. According to a study forecasting the impact of climate change on *Aedes* mosquitoes, climate change is expected to further expand the number of countries and regions at risk of dengue in the future, as the geographic area with optimal temperatures for *Aedes* mosquitoes expands [11]. Furthermore, altitude is an important environmental factor in dengue transmission because of its association with vector habitats [12]. A study from Nepal reported that altitude was negatively associated with dengue incidence [13].

Despite the urgent need to combat dengue, particularly in endemic areas, to the best of our knowledge, no nationwide epidemiological studies have been conducted on the association between dengue and meteorological factors in Lao PDR. Many studies for dengue in this region have focused primarily on serological studies rather than meteorological factors [14, 15]. Moreover, no studies have investigated whether or not these associations differ with altitude in Lao PDR. Focusing on Lao PDR, this study was aimed at estimating the association between meteorological factors (i.e., temperature and rainfall) and dengue transmission, and the potential effect modification by altitude.

## Materials and methods

### Data collection

#### Health data

Lao PDR has 18 jurisdictions, comprising 17 provinces and the capital city of Vientiane. These 18 jurisdictions are hereinafter referred to as “provinces”. We obtained weekly dengue incidence from all 1,281 health care facilities (HCFs) in Lao PDR from January 2015 to December 2019 from the World Health Organization Country Office of Lao PDR (Figure S1). We aggregated the number of dengue cases in multiple HCFs to obtain the weekly dengue incidence at the province level (Table 1 and Figure S2). To determine the association between meteorological factors and dengue incidence more precisely, the province of Houaphan was excluded from the statistical analysis because of the low number of reported dengue cases. Annual population data from 2015 to 2019 were obtained from the Lao Statistics Bureau [16].

**Table 1:** Descriptive statistics for dengue cases, weekly temperature and rainfall, and altitude by province from 2015 to 2019

Province	Number of HCFs	Total dengue cases (2015–2019)	Weekly dengue cases	Mean temperature (ºC)	Weekly total rainfall (mm)	Altitude of HCFs (m)
			Median (IQR)	Mean (SD)	Median (IQR)	Median (IQR)
Attapu	39	4749	12 (24)	28.8 (1.8)	7.3 (59.6)	116.0 (384.0)
Bokeo	47	155	0 (0)	26.0 (2.8)	8.3 (46.0)	397.5 (174.0)
Bolikhamxai	51	2868	4 (13)	26.9 (2.5)	11.4 (77.2)	232.0 (380.0)
Champasak	95	4284	5 (23)	28.3 (2.0)	10.0 (57.6)	107.0 (59.5)
Houaphan	86	5	0 (0)	21.4 (3.9)	8.1 (39.4)	850.0 (622.0)
Khammouan	104	5060	3 (10)	27.6 (2.6)	11.0 (65.2)	171.5 (45.3)
Louangnamtha	47	2059	0 (7)	24.1 (3.2)	11.5 (39.2)	718.0 (273.5)
Louangphabang	97	1970	2 (6)	26.5 (3.2)	9.1 (44.1)	503.0 (484.0)
Oudomxai	63	515	0 (1)	23.2 (3.3)	9.2 (37.9)	733.0 (314.0)
Phongsali	58	68	0 (0)	20.3 (3.6)	20.5 (50.4)	799.0 (393.5)
Salavan	82	3834	2 (12)	27.6 (2.3)	10.4 (58.6)	223.0 (273.3)
Savannakhet	178	8067	2 (19)	27.3 (2.8)	5.2 (43.2)	168.0 (45.0)
Vientiane	61	144	0 (0)	27.0 (2.7)	12.6 (67.2)	229.0 (84.5)
Vientiane Capital	53	18275	24.5 (67)	28.0 (2.5)	9.6 (43.3)	171.0 (10.0)
Xainyabouli	92	1225	0 (2)	26.4 (2.8)	11.2 (43.0)	438.5 (249.5)
Xaisomboun	26	450	0 (0)	20.6 (2.6)	24.0 (56.7)	418.0 (673.0)
Xekong	36	1750	2 (4.3)	27.8 (2.2)	11.5 (43.4)	775.5 (758.8)
Xiangkhouang	66	83	0 (0)	21.5 (3.3)	14.0 (45.0)	1112.0 (350.0)

*HCF* healthcare facility, *IQR* interquartile range, *SD* standard deviation

#### Meteorological data

Lao PDR is located in northern Southeast Asia and has a tropical monsoon climate with rainy and dry seasons. Daily data for the maximum and minimum temperatures and daily total rainfall January 2015 to December 2019 for each province were obtained from the Department of Meteorology and Hydrology, Department of Natural Resources and Environment, Lao PDR. The meteorological data for each province were obtained from a weather station in the provincial capital city. We calculated the averages from the daily data for the maximum and minimum temperatures and aggregated them into weekly data. We obtained daily relative humidity and wind speed for two most populous provinces (Vientiane Capital and Savannakhet) for sensitivity analysis form the Automated Surface Observing Systems (https://mesonet.agron.iastate.edu/request/download.phtml?network=LA__ASOS) and we aggregated them into weekly data. Available data periods for relative humidity and wind speed were January 2015 to December 2019 for Vientiane Capital and November 2015 to December 2019 for Savannakhet.

#### Altitude data

We obtained altitude data based on the location of the HCFs. The geographical coordinates of each HCF are publicly available on the Health Facility Master List Online (https://hfml.la). We calculated the median altitude across the HCFs by province and dichotomized the HCF altitude by the median value (low vs. high) for each province. We used the “getData” function of the “raster” package in the R software program to extract the geocoordinates.

#### Statistical analysis

Dengue outbreaks are caused by complex relationships between meteorological factors, such as temperature, rainfall, and vector ecology. These relationships are non-linear and exhibit delayed effects [17, 18]. We applied a flexible modeling approach using a distributed lag nonlinear model (DLNM) [19] to account for nonlinear and delayed associations between meteorological factors and dengue incidence using cross-basis functions.

The analysis was conducted in two stages. In the first stage, we applied a quasi-Poisson DLNM to estimate the associations between meteorological factors (i.e., weekly mean temperature and total rainfall) and weekly dengue incidence by province. We used the cross-basis function with the natural cubic B-spline (NCS) with one internal knot placed at the 50^th^ percentile of the meteorological factors in the exposure–response dimension and NCS with two equally spaced internal knots on the log scale in the lag–response dimension for temperature and rainfall. Based on previous studies [20], we extended the maximum lags to 22 and 12 weeks for temperature and rainfall, respectively. The calendar year was included as an indicator variable in the model to control for long-term trends. We also added log-transformed dengue cases from the previous week to reduce serial autocorrelation in the residuals. Model selection was guided by the Quasi-Akaike Information Criterion (QAIC) and the sum of the QAICs across provinces. Details are described in the sensitivity analysis section.

In the second stage, we applied a multivariate meta-regression model [21] to pool the province-specific estimates from the first stage and obtained national average estimates of the associations between meteorological factors and dengue incidence in 17 provinces. To explore the potential drivers of residual heterogeneity across provinces, we considered four potential meta-predictors, including provincial averages of weekly mean temperature and weekly total rainfall over the study period, along with latitude and altitude for each province. We fitted the model using the simplest manner with a single variable at a time, building up to a full model including all four variables, performed a multivariate Cochran’s Q test, and calculated the *I*^*2*^ statistics [22]. However, none of the potential meta-predictors significantly reduced the amount of residual heterogeneity (Table S1). The likelihood ratio tests for each meta-predictor also showed no evidence of effect modification of the predictors on the association between dengue and temperature or rainfall (*p* > 0.05) (Table S1). Nevertheless, we observed that the pooled cumulative relative risk (RR) for temperature changed in the model with mean temperature, compared to when other predictors were used (Table S1), suggesting that mean temperature may confound the pooled estimates. Hence, we a priori included the provincial mean temperature in the second-stage main model. We also calculated the best linear unbiased prediction (BLUP) to obtain province-specific association curves [23].

Furthermore, we conducted a separate analysis by including the dichotomized altitudes (low vs. high) for each province in the meta-regression model to explore how the associations between meteorological factors and dengue differed by altitude.

The association between meteorological variables and dengue was modeled using the “dlnm” and “mixmeta” packages [24]. All statistical analyses were performed using the R software (version 4.1.3).

#### Sensitivity analysis

Six additional conditions were applied in the sensitivity analysis (Table S2). First, we excluded the autocorrelation term (i.e., log-transformed dengue cases in the previous week) from the main model. Second, we altered the number of internal knots of the NCS from one to two and three for temperature and rainfall, respectively. Finally, we incorporated a week-of-year (WOY) variable into the model to adjust for seasonality using NCS with varying degrees of freedom of two, four, and six in each model. After adding WOY with degrees of freedom of four and six to the model, the QAIC for Xaisomboun (a smaller province with few dengue cases) became considerably inflated (Table S3). Therefore, we calculated the sum of the QAICs for 16 provinces (excluding Houaphan and Xaisomboun). Furthermore, we conducted a sensitivity analysis after adjusting for relative humidity and wind speed for Vientiane Capital and Savannakhet. Relative humidity and wind speed were incorporated as five-month moving-averages into the model using natural cubic B-spline with three degrees of freedom, respectively.

## Results

### Temporal and spatial patterns of dengue incidence in Lao PDR

**Fig. 1:**
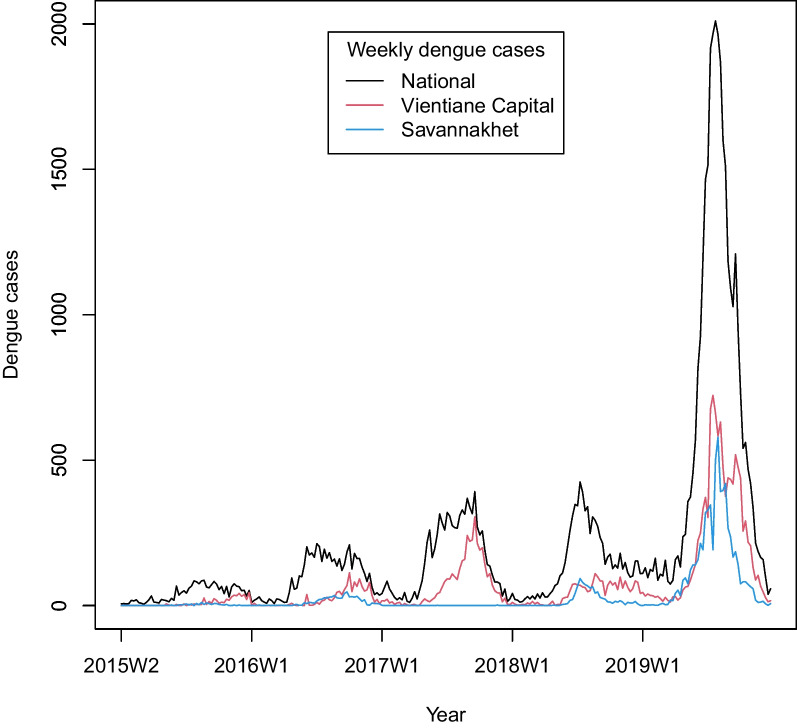
Time series plots of weekly dengue cases at the national level (black line) and the two most populous provinces, Vientiane Capital (red line) and Savannakhet (blue line), from 2015 to 2019

**Fig. 2:**
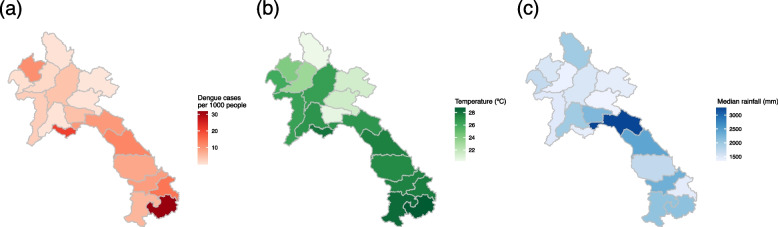
Five-year (2015–2019) spatial distribution of each variable: (**a**) population-adjusted dengue risk calculated by dividing the total dengue cases detected in each province by the average population over the same period; (**b**) mean weekly temperature by province (ºC); and (**c**) median total annual rainfall (mm) by province

A total of 55,561 dengue cases were reported in Lao PDR from 2015 to 2019. Figure 1 shows the weekly number of dengue cases at the national level in the two most populous provinces (Vientiane Capital and Savannakhet) during the study period. Generally, dengue outbreaks occurred between May and October. This period generally coincided with the rainy season in Lao PDR. The peak dengue incidence varied by region and year except for a nationwide outbreak in 2019 (Fig. 1 and S2). Figure 2a shows a risk map of dengue incidence per 1,000 population over the 5-year period. The incidence was high in Vientiane Capital and the central to southern regions, with the southern province of Attapu at a particularly high risk.

### Temporal and spatial patterns of weather in Lao PDR

Table 1 shows the descriptive statistics for the weekly mean temperature and total rainfall by province. The weekly mean temperature ranged from 20.3ºC (Phongsali) to 28.8ºC (Attapu), and the median weekly total rainfall ranged from 5.2 (Savannakhet) to 24.0 (Xaisomboun) mm. Figure 2b and c shows the spatial distributions of the mean temperature and annual rainfall from 2015 to 2019, respectively. In general, the southern provinces tended to be warmer than the northern ones. Although the annual rainfall varied widely by region and year, the greatest rainfall across all provinces was observed in the middle of the year, from June to October, during the study period. The distribution of rainfall in Lao PDR was heavily skewed to the right (i.e., with many days of 0 mm or almost no rainfall). The national average percentage of days with 0 mm of rainfall over the 5-year period was 64.8% (Table S4).

### Association of weekly mean temperature and total rainfall with the dengue incidence in Lao PDR

**Fig. 3:**
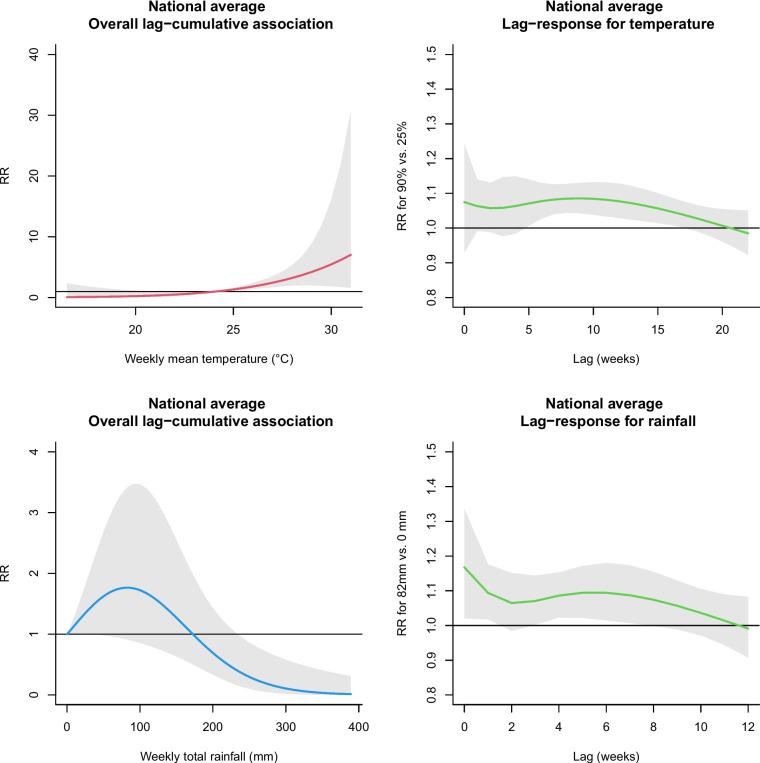
National average estimates of associations between dengue and two meteorological indicators over 22 weeks for temperature and 12 weeks for rainfall were pooled across 17 provinces (excluding Houaphan) in Lao PDR. Estimates were calculated using a distributed lag nonlinear model adjusted for the calendar year and log-transformed previous dengue cases. The solid line represents the estimated spline curve, and the shaded region indicates the 95% confidence interval. References in the overall lag–cumulative association curve are at 24 °C (25th percentile) for temperature and 0 mm for rainfall

Figure 3 shows the pooled national estimates of the weather–dengue associations and lag–response patterns for temperature and rainfall. The cumulative RR over lag weeks was estimated at 4.21 (95% confidence interval [CI]: 2.00–8.84) for a weekly mean temperature of 29 °C (90^th^ percentile) versus 24 °C (25^th^ percentile). Lagged effects of temperature were found for midterm lags (5–17 weeks). The association between weekly total rainfall and dengue showed an inverse U-shaped curve with a lag-cumulative RR for rainfall peaking at 82 mm (RR = 1.76, 95% CI: 0.91–3.40) versus no rain (0 mm) and decreased thereafter. 82 mm is about the 85^th^ percentile of total weekly rainfall, which is close to the 90^th^ percentile (103 mm) and can be considered a relatively large amount of rainfall. A protective effect was observed when rainfall exceeded 200 mm. RRs for moderate rainfall at 82 mm decreased with a longer lag.

**Fig. 4:**
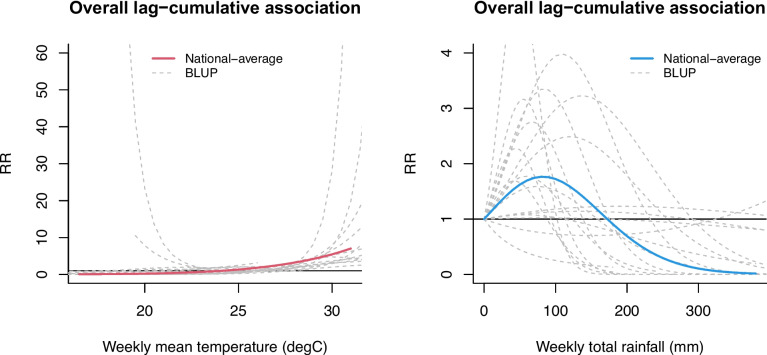
National average estimates and province-specific best linear unbiased predictions (BLUP) for overall lag-cumulative associations between dengue and meteorological indicators over 22 weeks for temperature and 12 weeks for rainfall were derived from a multivariate meta-regression analysis across 17 provinces (excluding Houaphan) in Lao PDR. Estimates were calculated using a distributed lag nonlinear model adjusted for the calendar year and log-transformed previous dengue cases. The solid line shows the estimated national average BLUP, and the dotted lines show the province-specific BLUP

Figure 4 shows the pooled national estimates and province-specific BLUPs for the lag-cumulative weather–dengue associations derived from the meta-regression model.

**Fig. 5:**
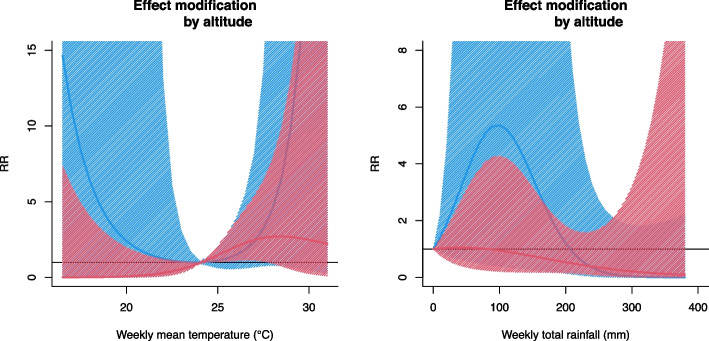
The average estimates of associations between dengue and two meteorological indicators by altitude across 17 provinces (2015–2019). Estimated associations between dengue and two meteorological indicators by altitude across 17 provinces (i.e., excluding Houaphan) (2015–2019). The solid curve lines are the average estimates from meta-regression for low (red line) and high (blue line) altitude for temperature (left) and rainfall (right), while the shaded lines show their 95% confidence intervals

The province-specific BLUP association curves show similar patterns among several provinces. However, the magnitude of the cumulative RRs varied across provinces, and the 95% confidence intervals also had a considerable range in variability (see also Table S5). The multivariate Cochran Q-tests and *I*^*2*^ statistics suggest a large amount of residual heterogeneity (*p* < 0.001 for the Cochran Q test, *I*^*2*^ = 69.3% for the association with temperature; *p* = 0.006 for the Cochran Q test, *I*^*2*^ = 43.7% for the association with rainfall). We observed that the residual heterogeneity changed little even after adding the meta-predictors to the model, i.e., province-level temperature, rainfall, latitude, and altitude (Table S1). We found no evidence of effect modification by altitude on the weather–dengue associations (Fig. 5).

### Sensitivity analysis

The sum of the QAICs significantly increased after excluding the autocorrelation term relative to the main model (Table S2, Model 1), suggesting that the model accounting for autocorrelation had a better fit than the model without the term. For the models with different numbers of internal knots of NCS for both temperature–dengue and rainfall–dengue associations, the sum of QAICs changed little with the varying number of knots in NCS (Table S2, models 2 and 3), and the cumulative association between temperature and rainfall showed a wider 95% CI than the main model. Finally, a sensitivity analysis of 16 provinces (excluding Houaphan and Xaisomboun) showed no significant difference in the sum of QAICs between the seasonality-adjusted models and the main model (Table S2, models 4–6).

Table S6 shows the descriptive statistics for the weekly relative humidity and wind speed and results of sensitivity analysis controlling for them in the modeling. There was no significant difference in the association between dengue and temperature or rainfall with the adjustment of relative humidity and wind speed in Vientiane Capital. On the other hand, in Savannakhet, the association between dengue and temperature after adjusting for wind speed showed higher relative risk with a larger 95% confidence interval than without adjustment (Table S6). Furthermore, we found that adjustment for wind speed increased the uncertainty of the estimate, especially for shorter lag weeks (from current to 10 weeks).

## Discussion

This is the first nationwide epidemiological study conducted on the association between meteorological factors and dengue incidence in the context of nonlinear and delayed associations in Lao PDR. While many studies have been conducted in Southeast Asia and around the world on the association between factors such as temperature and rainfall and the dengue incidence, our study provides useful insight on the major environmental determinants of dengue in Lao PDR. We observed that the risk of dengue increased with weekly mean temperature. The association for weekly total rainfall showed an inverse U-shaped pattern, in which moderate rainfall presented the highest dengue risk and excessive rainfall was protective. However, the association between meteorological factors and dengue incidence was heterogeneous across provinces. Weekly mean temperature, weekly total rainfall, latitude, and altitude could not explain the residual heterogeneity in this study. Further investigation is required to assess other factors, such as local characteristics, that may be able to explain this heterogeneity.

The results of this study showed that the risk of dengue incidence increases with rising temperatures, generally consistent with previous studies [25–29]. Temperature also affects the life cycle of mosquitoes [30]. Higher temperatures accelerate the rate of dengue virus replication in mosquitoes and shorten the extrinsic incubation period [6], which may increase in dengue transmission, thereby increasing the number of dengue cases. A delayed effect of approximately 5–17 weeks was observed in the association between temperature and dengue in the present study. A modeling study from Vietnam [31] found that heat waves exert a delayed effect on dengue outbreaks and that the delayed effect differed with the magnitude of the outbreak. Heat waves exerted a longer delayed effect on large outbreaks than on small or medium outbreaks. Moreover, our results demonstrating considerable heterogeneity across regions are consistent with the findings of a previous study on severe dengue cases and temperatures conducted in Thailand [29].

Previous studies have reported a positive association between dengue and rainfall [25, 32]. However, we found an inverse U-shaped relationship between rainfall and dengue, with extremely heavy rainfall reducing the risk of dengue. A potential explanation for this may be that heavy rainfall washed away mosquito breeding sites, thereby affecting the vector population. The association between mosquito-borne diseases and extreme rainfall and flooding is complex, with varying results among studies; however, several studies have similarly reported that extremely heavy rainfall decreases the risk of dengue [33, 34]. A recent review of 45 dengue studies conducted worldwide [18] found that, after flooding events, there was a temporary decrease (after less than a month), followed by an increase (after one to four months) in dengue incidence. A modeling study from Barbados incorporating nonlinear and delayed effects of the weather–dengue association [17] showed that excess rainfall increased the risk at shorter lead times (between one and two months), while drought conditions were positively associated with risk at longer lead times (up to 5 months).

This study showed no evidence of effect measurement modification by altitude. However, a study from Nepal [13] reported a higher dengue risk at lower altitudes and a lower risk at higher altitudes. A study from Kenya [35] on malaria, a mosquito-borne disease similar to dengue, found a positive correlation between rainfall and malaria in the lowlands, but no correlation in the highlands. Studies from the Americas [36] have shown that high altitudes limit the occurrence of *Aedes* mosquitoes. A possible reason why altitude did not modify the weather–dengue association in this study is that people in mountainous areas in Lao PDR use containers without lids for water storage [37], thus providing breeding sites for the vectors. The World Health Organization (WHO) reports that large water jars or cement tanks make up 80% of containers found with *A. aegypti* larvae in Lao PDR [38]. A study from Indonesia [39] suggested that large, open cement tanks for water storage are the most favorable larval habitat at high altitudes. The presence of open water storage in elevated areas may have masked the effect of altitude limiting the habitat of mosquitoes in Lao PDR.

This study has several strengths. First, to our knowledge, this was the first study in Lao PDR to use nationwide data to examine the spatiotemporal patterns of dengue epidemics. This allowed us to determine the association between meteorological factors and dengue incidence across the country. Second, the developed model accounted for the nonlinear and delayed effects of temperature and rainfall on dengue incidence. Third, our analysis demonstrated the heterogeneity in risk patterns at the local level, providing evidence to aid resource allocation, thereby contributing to future dengue control measures.

However, this study has some limitations. First, the lack of entomological data with respect to the dengue vector meant that we could not examine how the vector distribution affected the association between meteorological factors and the incidence of dengue. Second, no data were available on the diagnostic method, viral serotypes, patient age, or dengue severity. Thus, there may have been over- or under-reporting of cases [40]. Population-level serotype shifts vary yearly, thereby affecting population immunity [41]. For example, a study from northeastern Thailand [42] showed that dengue generally tends to be prevalent among young people. However, in recent years, the number of infections among the elderly has increased. A study from Vietnam [43] found that older people are more likely to develop symptomatic disease than younger people. Thus, the age of patients with dengue may have implications for the extent to which the national health surveillance system can capture cases within the population. Further studies are required to determine how entomological, serological, and demographic data, which could not be ascertained in this study, influence the association between meteorological factors and dengue.

The main approach to preventing dengue is vector control, owing to the lack of vaccines and commercially available specific antivirals for dengue [44]. Recently, early warning systems (EWSs) have been developed worldwide as effective countermeasures against the spread of dengue [45]. The associations between meteorological factors and dengue incidence in this study may contribute to the development of a climate-based EWS, which may help health managers make decisions, particularly in Lao PDR and neighboring countries, where human and material resources are limited. However, these associations could be affected by regional characteristics; thus, longer periods of more granular data are required to contribute to the development of precise EWSs, particularly in less populated areas of Lao PDR.

## Conclusions

The risk of dengue incidence in Lao PDR is seasonal and varies by region and year. In this study, we found that dengue risk increases with increasing temperatures and moderate rainfall, but decreases with extreme rainfall in Lao PDR. Regional heterogeneity also exists in the association of temperature and rainfall with dengue in Lao PDR. We found no evidence that altitude is an effect measure modifier of the weather–dengue relationship. Further studies are required to better understand this pattern using data collected over longer time periods. The associations between meteorological factors and dengue incidence in Lao PDR obtained in this study may provide a useful scientific basis for EWSs and climate-informed dengue control policies appropriate for this region.

### Supplementary Information

**Additional file 1.** 

## Data Availability

The data used in this study were obtained from the World Health Organization (WHO) with permission to use only for this study. The data may be accessible to others by requesting the data access to WHO via the corresponding author.

## Abbreviations

Lao PDR: Lao People’s Democratic Republic
HCFs: Health care facilities
DLNM: Distributed lag nonlinear model
NCS: Natural cubic B-spline
QAIC: Quasi-Akaike Information Criterion
BLUP: Best linear unbiased prediction
WOY: Week-of-year
RR: Relative risk
CI: Confidence Interval
EWSs: Early warning systems

## References

[1] Messina JP (2019). The current and future global distribution and population at risk of dengue. Nat Microbiol.

[2] World Health Organization. Dengue and severe dengue. 2022.

[3] Lambrechts L, Scott TW, Gubler DJ (2010). Consequences of the expanding global distribution of Aedes albopictus for dengue virus transmission. PLoS Negl Trop Dis.

[4] Tian N, et al. Dengue incidence trends and its burden in major endemic regions from 1990 to 2019. Trop Med Infect Dis. 2022;7(8):180. 10.3390/tropicalmed7080180 PMC9416661 36006272

[5] Hung TM (2020). Productivity costs from a dengue episode in Asia: a systematic literature review. BMC Infect Dis.

[6] Morin CW, Comrie AC, Ernst K (2013). Climate and dengue transmission: evidence and implications. Environ Health Perspect.

[7] Nik Abdull Halim N.M.H., et al. A systematic review and meta-analysis of the effects of temperature on the development and survival of the Aedes mosquito. Front Public Health. 2022; 10: 1074028. 10.3389/fpubh.2022.1074028 PMC9806355 36600940

[8] Regis LN (2014). Characterization of the spatial and temporal dynamics of the dengue vector population established in urban areas of Fernando de Noronha, a Brazilian oceanic island. Acta Trop.

[9] Wang Y (2022). Impact of extreme weather on dengue fever infection in four Asian countries: a modelling analysis. Environ Int.

[10] Choi Y (2016). Effects of weather factors on dengue fever incidence and implications for interventions in Cambodia. BMC Public Health.

[11] Ryan SJ (2019). Global expansion and redistribution of Aedes-borne virus transmission risk with climate change. PLoS Negl Trop Dis.

[12] Lozano-Fuentes S (2012). The dengue virus mosquito vector Aedes aegypti at high elevation in Mexico. Am J Trop Med Hyg.

[13] Gyawali N (2021). Patterns of dengue in Nepal from 2010–2019 in relation to elevation and climate. Trans R Soc Trop Med Hyg.

[14] Doum D, et al. Dengue Seroprevalence and Seroconversion in Urban and Rural Populations in Northeastern Thailand and Southern Laos. Int J Environ Res Public Health. 2020;17(23):9134. 10.3390/ijerph17239134 PMC7731008 33297445

[15] Calvez E, et al. Trends of the dengue serotype-4 circulation with epidemiological, phylogenetic, and entomological insights in Lao PDR between 2015 and 2019. Pathogens. 2020;9(9):728. 10.3390/pathogens9090728 PMC7557816 32899416

[16] Lao Statistics Bureau. 2021.

[17] Lowe R (2018). Nonlinear and delayed impacts of climate on dengue risk in Barbados: a modelling study. PLoS Med.

[18] Coalson JE (2021). The complex epidemiological relationship between flooding events and human outbreaks of mosquito-borne diseases: a scoping review. Environ Health Perspect.

[19] Gasparrini A, Armstrong B, Kenward MG (2010). Distributed lag non-linear models. Stat Med.

[20] Wang P (2021). A systematic review on lagged associations in climate-health studies. Int J Epidemiol.

[21] Sera F, Gasparrini A (2022). Extended two-stage designs for environmental research. Environ Health.

[22] Higgins JP, Thompson SG (2002). Quantifying heterogeneity in a meta-analysis. Stat Med.

[23] Gasparrini A, Armstrong B, Kenward MG (2012). Multivariate meta-analysis for non-linear and other multi-parameter associations. Stat Med.

[24] Gasparrini A (2011). Distributed Lag Linear and Non-Linear Models in R: The Package dlnm. J Stat Softw.

[25] Johansson MA, Dominici F, Glass GE (2009). Local and global effects of climate on dengue transmission in Puerto Rico. PLoS Negl Trop Dis.

[26] Lu L (2009). Time series analysis of dengue fever and weather in Guangzhou, China. BMC Public Health.

[27] Wu X (2018). Non-linear effects of mean temperature and relative humidity on dengue incidence in Guangzhou, China. Sci Total Environ.

[28] Kakarla SG (2019). Lag effect of climatic variables on dengue burden in India. Epidemiol Infect.

[29] Xu Z (2019). Spatiotemporal patterns and climatic drivers of severe dengue in Thailand. Sci Total Environ.

[30] Naish S (2014). Climate change and dengue: a critical and systematic review of quantitative modelling approaches. BMC Infect Dis.

[31] Cheng J (2020). Heatwaves and dengue outbreaks in Hanoi, Vietnam: New evidence on early warning. PLoS Negl Trop Dis.

[32] Wongkoon S, Jaroensutasinee M, Jaroensutasinee K (2013). Weather factors influencing the occurrence of dengue fever in Nakhon Si Thammarat Thailand. Trop Biomed.

[33] Ehelepola ND (2015). A study of the correlation between dengue and weather in Kandy City, Sri Lanka (2003–2012) and lessons learned. Infect Dis Poverty.

[34] Iguchi JA, Seposo XT, Honda Y (2018). Meteorological factors affecting dengue incidence in Davao, Philippines. BMC Public Health.

[35] Matsushita N, et al. Differences of rainfall-malaria associations in lowland and highland in Western Kenya. Int J Environ Res Public Health. 2019;16(19):3693. 10.3390/ijerph16193693 PMC6801446 31575076

[36] Watts AG (2017). Elevation as a proxy for mosquito-borne Zika virus transmission in the Americas. PLoS One.

[37] Vannavong N (2017). Effects of socio-demographic characteristics and household water management on Aedes aegypti production in suburban and rural villages in Laos and Thailand. Parasit Vectors.

[38] World Health Organization. Managing regional public goods for health: community-based dengue vector control 2013. 2013.

[39] Sayono S (2017). Altitudinal distribution of Aedes indices during dry season in the dengue endemic area of Central Java Indonesia. Ann Parasitol.

[40] Khampapongpane B (2014). National dengue surveillance in the Lao People's Democratic Republic, 2006–2012: epidemiological and laboratory findings. Western Pac Surveill Response J.

[41] Bowman LR (2016). Alarm variables for dengue outbreaks: a multi-centre study in Asia and Latin America. PLoS One.

[42] Phanitchat T (2019). Spatial and temporal patterns of dengue incidence in northeastern Thailand 2006–2016. BMC Infect Dis.

[43] Thai KT (2011). Age-specificity of clinical dengue during primary and secondary infections. PLoS Negl Trop Dis.

[44] Qian X, Qi Z. Mosquito-borne flaviviruses and current therapeutic advances. Viruses. 2022;14(6):1226. 10.3390/v14061226 PMC9229039 35746697

[45] Hussain-Alkhateeb L (2021). Early warning systems (EWSs) for chikungunya, dengue, malaria, yellow fever, and Zika outbreaks: What is the evidence? A scoping review. PLoS Negl Trop Dis.

