# Supplementary material for: Association between environmental factors and dengue incidence in Lao People’s Democratic Republic: a nationwide time-series study
Source: BMC Public Health. 2023 Nov 27;23:2348. doi: 10.1186/s12889-023-17277-0 (PMC10683213; doi:10.1186/s12889-023-17277-0)
Supplement: Supplementary file 1 — Additional file 1. [file 12889_2023_17277_MOESM1_ESM.docx]

**Supplemental Information**

**Association between environmental factors and dengue incidence in the Lao People’s Democratic Republic: a nationwide time-series study**

Masumi Sugeno^1^, Erin C. Kawazu^2^, Hyun Kim^3^, Virasack Banouvong^4^, Nazife Pehlivan^5^, Daniel Gilfillan^6^, Ho Kim^5*^, Yoonhee Kim^1*^

^1^ Department of Global Environmental Health, Graduate School of Medicine, The University of Tokyo, Tokyo, Japan

^2^ Institute for Global Environmental Strategies, Hayama, Japan

^3^ School of Public Health, University of Minnesota Twin Cities, Minneapolis, US

^4^ Lao PDR Centre for Malariology, Parasitology and Entomology, Vientiane Capital, Lao People's Democratic Republic

^5^ Graduate School of Public Health, Seoul National University, Seoul, Republic of Korea

^6^ Fenner School of Environment and Society, the Australian National University, Canberra, Australian Capital Territory, Australia

**Table of contents**

Model framework3

Table S1. Cumulative relative risk, Cochran’s Q test for heterogeneity (p-value), *I^2^* statistic (%), and likelihood ratio tests in different multivariate meta-regression models 4

Table S2. The sum of QAIC for model selection5

Table S3. Results of sensitivity analysis at the province level for the main model and the model with seasonality terms7

Table S4. Descriptive statistics for weekly temperature, rainfall, and dengue risk by province over five years (2015-2019)8

Table S5. Province-specific cumulative relative risk obtained from BLUP10

Table S6. Descriptive statistics for relative humidity and wind speed and results of sensitivity analysis controlling for relative humidity and wind speed in Vientiane Capital and Savannakhet12

Figure S1. Locations of geocoded HCFs (n = 1,142) and cumulative dengue cases detected at each facility13

Figure S2. Time-series plots of weekly dengue cases in 18 provinces over five years (2015-2019)14

**Model framework**

We used a distributed lag nonlinear model (DLNM) with a Poisson distribution allowing for overdispersion. The regression model is represented as follows:

$$y_{t} \sim Poisson (\lambda_{t})$$

$$\text{Log}\left[ \text{E(}\lambda_{t}) \right]=\alpha_{0}+f\left( \boldsymbol{T}_{t};\beta_{1} \right)+f\left( \boldsymbol{R}_{t};\beta_{2} \right)+y_{t-1}\theta+\text{year}_{t}\alpha_{j}$$

where $y_{t}$ is the observed dengue cases on week t.

$f\left( \boldsymbol{T}_{t};\beta_{1} \right)$ represents a flexible function formulated with a cross-basis to describe nonlinear and nonlinearly-delayed association between temperature and dengue. $\boldsymbol{T}_{t}={(T}_{0}, T_{t-1}, \ldots, T_{t-L})$ is a vector representing weekly mean temperature on week *t* and over the previous L weeks.

$f\left( \boldsymbol{R}_{t};\beta_{1} \right)$ represents a flexible function formulated with a cross-basis to describe nonlinear and nonlinearly-delayed association between rainfall and dengue. $\boldsymbol{R}_{t}={(R}_{0}, R_{t-1}, \ldots, R_{t-L})$ is a vector representing weekly total rainfall on week *t* and over the previous L weeks.

$y_{t-1}\theta$ indicates log-transformed dengue cases in the preceding week (*t*-1).

$\alpha_{0}$ is an intercept.

$\alpha_{j}$ (j=1, …., 5) is a coefficient for $\text{year}_{t}$ to capture the long-term changes in unmeasured time-varying confounders.

Table S1. Cumulative relative risk, Cochran’s Q test for heterogeneity (p-value), *I^2^* statistic (%), and likelihood ratio tests in different multivariate meta-regression models

|  |  | Temperature | | | | Rainfall | | | |  |  |
| --- | --- | --- | --- | --- | --- | --- | --- | --- | --- | --- | --- |
| Model | Predictor | Cumulative RR^*^  (95% CI) | Q test^*^ | *I^2*^* | *p* for LRT | Cumulative RR^*^  (95% CI) | Q test^*^ | *I^2*^* | *p* for LRT |  |  |
| Intercept - only | - | 5.12 (2.61–10.04) | p < 0.001 | 68.1% |  | 1.75 (1.04–2.97) | p = 0.007 | 42.1% |  |  |  |
| Single predictor | Weekly mean temperature  (same as in the main model) | 4.21 (2.00–8.84) | p < 0.001 | 69.3% | 0.428 | 1.76 (0.91–3.40) | p = 0.006 | 43.7% | 0.243 |  |  |
|  | Weekly total rainfall | 4.90 (2.43–9.89) | p < 0.001 | 68.5% | 0.590 | 1.90 (1.16–3.10) | p = 0.01 | 41.3% | 0.313 |  |  |
|  | Latitude | 4.68 (2.26–9.68) | p < 0.001 | 69.3% | 0.885 | 1.70 (0.94–3.07) | p = 0.004 | 45.2% | 0.329 |  |  |
|  | Altitude | 4.91 (2.38–10.14) | p < 0.001 | 69.6% | 0.511 | 1.79 (0.99–3.25) | p = 0.009 | 41.3% | 0.890 |  |  |
| Two predictors | Temperature, Rainfall | 4.25 (1.96–9.20) | p < 0.001 | 70.3% |  | 1.80 (0.95–3.42) | p = 0.008 | 42.8% |  |  |  |
| Three predictors | Temperature, Rainfall, Altitude | 4.06 (1.83–9.05) | p < 0.001 | 71.3% |  | 1.77 (0.91–3.46) | p = 0.009 | 43.7% |  |  |  |
| Full model | Temperature, Rainfall, Latitude, Altitude | 4.06 (1.73–9.54) | p < 0.001 | 72.5% |  | 1.92 (0.93–3.96) | p = 0.012 | 43.5% |  |  |  |

^*^RR = relative risk; CI = confidence interval.

^*^Q test : The p-value is an indication of the extent of between-study variability.

^*^*I^2^* : Proportion of total variation in estimates attributable to heterogeneity across provinces.

Table S2. The sum of QAIC* for model selection

| Model | Autocorrelation  (AC) | Seasonality  (Week-of-year) | Number of internal knots | | Sum of QAIC*  for 17 provinces  (excluding Houaphan) | Sum of QAIC*  for 16 provinces  (excluding Houaphan and Xaisomboun) |
| --- | --- | --- | --- | --- | --- | --- |
|  |  |  | Temperature Rainfall | |  |  |
| Main model | With AC term | - | 1 knot at 50^th^ percentile | 1 knot at 50^th^ percentile | 30291.4 | 23028.9 |
| 1 | - | - | 1 knot at 50^th^ percentile | 1 knot at 50^th^ percentile | 37955.5 | 26818.5 |
| 2 | With AC term | - | 2 knots at 33^rd^ and 66^th^ percentiles | 2 knots at 50^th^ and 75^th^ percentiles | 24094.5 | 23295.4 |
| 3 | With AC term | - | 3 knots at 25^th^, 50^th^, and 75^th^ percentiles | 2 knots at 50^th^ and 75^th^ percentiles | 23651.7 | 23069.2 |
| 4 | With AC term | NCS* with df of 2 | 1 knot at 50^th^ percentile | 1 knot at 50^th^ percentile | 27683.4 | 22570.0 |
| 5 | With AC term | NCS* with df of 4 | 1 knot at 50^th^ percentile | 1 knot at 50^th^ percentile | 60493.1 | 21964.0 |
| 6 | With AC term | NCS* with df of 6 | 1 knot at 50^th^ percentile | 1 knot at 50^th^ percentile | 49306.4 | 22652.3 |

*QAIC = Quasi-Akaike Information Criterion

*NCS = natural cubic B-spline

Table S3. Results of sensitivity analysis at the province level for the main model and models with seasonality terms

| Province | Main model | NCS^*^ with df of 2 | NCS^*^ with df of 4 | NCS^*^ with df of 6 |
| --- | --- | --- | --- | --- |
| Attapu | 2162.5 | 2144.1 | 2154.6 | 2129.0 |
| Bokeo | 513.3 | 525.5 | 561.7 | 1088.5 |
| Bolikhamxai | 1632.6 | 1584.6 | 1575.8 | 1485.6 |
| Champasak | 2659.5 | 2658.7 | 2533.2 | 2537.6 |
| Houaphan | NA | NA | NA | NA |
| Khammouan | 1593.3 | 1556.4 | 1513.6 | 1494.3 |
| Louangnamtha | 1906.9 | 1941.0 | 1931.0 | 1943.0 |
| Louangphabang | 1452.6 | 1408.2 | 1418.8 | 1416.9 |
| Oudomxai | 621.5 | 608.4 | 614.7 | 586.3 |
| Phongsali | 267.8 | 261.7 | 259.8 | 263.7 |
| Salavan | 1880.7 | 1860.7 | 1795.1 | 1796.2 |
| Savannakhet | 1698.1 | 1664.5 | 1636.9 | 1849.3 |
| Vientiane | 377.5 | 319.0 | 233.9 | 226.1 |
| Vientiane Capital | 3653.0 | 3481.6 | 3238.5 | 3255.7 |
| Xainyabouli | 1134.1 | 1079.2 | 1058.5 | 1077.3 |
| Xaisomboun | 7262.4 | 5113.4 | 38529.1 | 26654.1 |
| Xekong | 1157.9 | 1157.8 | 1112.7 | 1097.9 |
| Xiangkhouang | 317.4 | 318.5 | 325.3 | 404.9 |

*NCS = natural cubic B-spline

Table S4. Descriptive statistics for weekly temperature, rainfall, and dengue risk by province over the study period (2015-2019)

| Province | Weekly mean temperature (ºC) | | | % days with 0 mm rainfall | Weekly total rainfall (by percentile; mm) | | | | | | | Weekly dengue cases (by percentile) | | | | | | |
| --- | --- | --- | --- | --- | --- | --- | --- | --- | --- | --- | --- | --- | --- | --- | --- | --- | --- | --- |
|  | Mean ± SD | Min | Max |  | 0th | 50th | 75th | 90th | 95th | 100th | IQR* | 0th | 50th | 75th | 90th | 95th | 100th | IQR* |
| Attapu | 28.8 ± 1.8 | 23.3 | 34.2 | 66.3 | 0.0 | 7.3 | 59.6 | 119.6 | 160.3 | 472.6 | 59.6 | 0.0 | 12.0 | 29.0 | 43.0 | 53.1 | 98.0 | 24.0 |
| Bokeo | 26.0 ± 2.8 | 16.3 | 32.2 | 65.1 | 0.0 | 8.3 | 46.0 | 97.4 | 117.7 | 194.0 | 46.0 | 0.0 | 0.0 | 0.0 | 1.0 | 2.1 | 21.0 | 0.0 |
| Bolikhamxai | 26.9 ± 2.5 | 16.7 | 32.6 | 60.2 | 0.0 | 11.4 | 77.2 | 187.8 | 230.4 | 851.3 | 77.2 | 0.0 | 4.0 | 14.0 | 27.1 | 47.0 | 97.0 | 13.0 |
| Champasak | 28.3 ± 2.0 | 21.2 | 34.1 | 69.1 | 0.0 | 10.0 | 57.6 | 117.0 | 149.9 | 449.5 | 57.6 | 0.0 | 5.0 | 24.0 | 46.1 | 68.1 | 208.0 | 23.0 |
| Houaphan | 21.4 ± 3.9 | 9.0 | 26.5 | 68.5 | 0.0 | 8.1 | 39.5 | 91.8 | 132.4 | 296.5 | 39.4 | 0.0 | 0.0 | 0.0 | 0.0 | 0.0 | 2.0 | 0.0 |
| Khammouan | 27.6 ± 2.6 | 19.1 | 34.4 | 63.2 | 0.0 | 11.0 | 65.2 | 134.0 | 201.2 | 500.6 | 65.2 | 0.0 | 3.0 | 11.0 | 46.2 | 106.2 | 279.0 | 10.0 |
| Louangnamtha | 24.1 ± 3.2 | 13.2 | 29.0 | 60.5 | 0.0 | 11.5 | 39.4 | 73.1 | 99.9 | 207.9 | 39.2 | 0.0 | 0.0 | 7.0 | 24.2 | 51.1 | 107.0 | 7.0 |
| Louangphabang | 26.5 ± 3.2 | 14.2 | 32.6 | 68.2 | 0.0 | 9.1 | 44.1 | 93.0 | 119.4 | 389.7 | 44.1 | 0.0 | 2.0 | 6.0 | 14.0 | 35.1 | 137.0 | 6.0 |
| Oudomxai | 23.2 ± 3.3 | 12.1 | 29.1 | 63.8 | 0.0 | 9.2 | 37.9 | 77.4 | 104.0 | 180.3 | 37.9 | 0.0 | 0.0 | 1.0 | 4.0 | 7.0 | 117.0 | 1.0 |
| Phongsali | 20.3 ± 3.6 | 7.0 | 27.8 | 57.2 | 0.0 | 20.5 | 51.9 | 94.0 | 119.0 | 274.0 | 50.4 | 0.0 | 0.0 | 0.0 | 1.0 | 2.0 | 4.0 | 0.0 |
| Salavan | 27.6 ± 2.3 | 19.3 | 33.9 | 64.3 | 0.0 | 10.4 | 58.6 | 110.7 | 188.4 | 647.8 | 58.6 | 0.0 | 2.0 | 12.0 | 23.0 | 64.6 | 306.0 | 12.0 |
| Savannakhet | 27.3 ± 2.8 | 18.1 | 34.7 | 76.0 | 0.0 | 5.2 | 43.2 | 95.2 | 134.4 | 434.7 | 43.2 | 0.0 | 2.0 | 19.0 | 75.5 | 185.4 | 579.0 | 19.0 |
| Vientiane | 27.0 ± 2.7 | 17.2 | 32.0 | 68.1 | 0.0 | 12.6 | 67.2 | 115.9 | 147.2 | 249.0 | 67.2 | 0.0 | 0.0 | 0.0 | 2.0 | 4.0 | 11.0 | 0.0 |
| Vientiane Capital | 28.0 ± 2.5 | 17.7 | 33.5 | 70.6 | 0.0 | 9.6 | 43.3 | 82.4 | 102.6 | 286.1 | 43.3 | 0.0 | 24.5 | 71.0 | 206.8 | 372.2 | 723.0 | 67.0 |
| Xainyabouli | 26.4 ± 2.8 | 15.4 | 32.0 | 65.5 | 0.0 | 11.2 | 43.0 | 71.8 | 101.5 | 186.7 | 43.0 | 0.0 | 0.0 | 2.0 | 14.0 | 27.0 | 81.0 | 2.0 |
| Xaisomboun | 20.6 ± 2.6 | 11.1 | 24.3 | 52.8 | 0.0 | 24.0 | 57.7 | 108.2 | 172.5 | 342.6 | 56.7 | 0.0 | 0.0 | 0.0 | 7.0 | 12.0 | 30.0 | 0.0 |
| Xekong | 27.8 ± 2.2 | 21.0 | 32.5 | 66.5 | 0.0 | 11.5 | 43.4 | 98.5 | 156.1 | 531.2 | 43.4 | 0.0 | 2.0 | 4.3 | 23.0 | 41.1 | 75.0 | 4.3 |
| Xiangkhouang | 21.5 ± 3.3 | 9.7 | 26.5 | 61.0 | 0.0 | 14.0 | 45.1 | 76.6 | 100.6 | 415.7 | 45.0 | 0.0 | 0.0 | 0.0 | 1.0 | 2.0 | 5.0 | 0.0 |

*IQR = Interquartile range

Table S5. Province-specific cumulative relative risk obtained from BLUP. The overall lag-cumulative relative risk for the 90^th^ percentile of weekly mean temperature relative to the 25^th^ percentile and the overall lag-cumulative relative risk for the 82mm of weekly total rainfall relative to no rain (0mm) are shown.

| Province | Total dengue cases  (2015–2019) | Cumulative RR^*^ for weekly mean temperature (95% confidence interval) | Cumulative RR^*^ for weekly total rainfall  (95% confidence interval) |
| --- | --- | --- | --- |
| Attapu | 4749 | 2.42 (0.96 - 6.07) | 1.20 (0.44 - 3.26) |
| Bokeo | 155 | 2.99 (0.83 - 10.8) | 0.88 (0.37 - 2.09) |
| Bolikhamxai | 2868 | 2.83 (1.27 - 6.31) | 0.99 (0.42 - 2.33) |
| Champasak | 4284 | 4.41 (1.69 - 11.49) | 2.49 (0.73 - 8.50) |
| Khammouan | 5060 | 7.81 (3.06 - 19.92) | 0.71 (0.22 - 2.25) |
| Louangnamtha | 2059 | 1.28 (0.26 - 6.44) | 3.62 (0.74 - 17.57) |
| Louangphabang | 1970 | 16.76 (5.12 - 54.80) | 1.04 (0.30 - 3.60) |
| Oudomxai | 515 | 2.19 (0.25 - 19.26) | 0.32 (0.06 - 1.58) |
| Phongsali | 68 | 1.78 (0.24 - 13.41) | 1.86 (0.11 - 31.16) |
| Salavan | 3834 | 10.75 (3.31 - 34.96) | 1.14 (0.38 - 3.36) |
| Savannakhet | 8067 | 4.68 (1.90 - 11.52) | 3.90 (1.44 -10.59) |
| Vientiane | 144 | 2.96 (0.65 - 13.48) | 1.14 (0.43 - 3.01) |
| Vientiane Capital | 18275 | 3.38 (1.22 - 9.38) | 1.58 (0.67 - 3.75) |
| Xainyabouli | 1225 | 4.43 (0.97 - 20.23) | 1.34 (0.40 - 4.54) |
| Xaisomboun | 450 | 1.16 (0.03 - 51.96) | 1.94 (0.11 - 34.85) |
| Xekong | 1750 | 10.01 (2.70 - 37.03) | 2.97 (1.22 - 7.19) |
| Xiangkhouang | 83 | 2.48 (0.10 - 60.61) | 3.32 (0.36 - 30.51) |

^*^RR = relative risk

Table S6. Descriptive statistics for relative humidity and wind speed and results of sensitivity analyses controlling for relative humidity and wind speed in Vientiane Capital and Savannakhet. The overall lag-cumulative relative risk for the 90^th^ percentile of weekly mean temperature relative to the 25^th^ percentile and the overall lag-cumulative relative risk for the 82 mm of weekly total rainfall relative to no rain (0 mm) are shown.

| Province | Relative humidity (%)  Mean (SD^*^) | Wind speed (mph)  Mean (SD^*^) | Model type | Cumulative RR^*^ for weekly mean temperature (95% confidence interval) | Cumulative RR^*^ for weekly total rainfall  (95% confidence interval) |
| --- | --- | --- | --- | --- | --- |
| Vientiane Capital | 71.4 (8.81) | 1.63 (0.30) | With RH | 2.16 (0.83 - 5.57) | 2.27 (0.72 - 7.14) |
|  |  |  | With WS | 2.64 (0.94 - 7.44) | 2.63 (0.98 - 7.06) |
|  |  |  | With RH and WS | 2.18 (0.81 - 5.90) | 2.55 (0.80 - 8.12) |
|  |  |  | Without RH or WS (same as in the main model) | 2.76 (1.03 - 7.40) | 2.53 (0.94 - 6.80) |
| Savannakhet | 66.1 (10.1) | 1.61 (0.50) | With RH | 6.92 (2.05- 23.38) | 1.43 (0.29 - 7.11) |
|  |  |  | With WS | 23.54 (2.72 - 203.42) | 2.39 (0.21 - 27.76) |
|  |  |  | With RH and WS | 20.09 (2.32 - 173.8) | 1.68 (0.11 - 24.43) |
|  |  |  | Without RH or WS (same as in the main model) | 5.25 (1.69 - 16.29) | 5.06 (1.35 - 18.94) |

^*^SD = standard deviation

^*^RR = relative risk


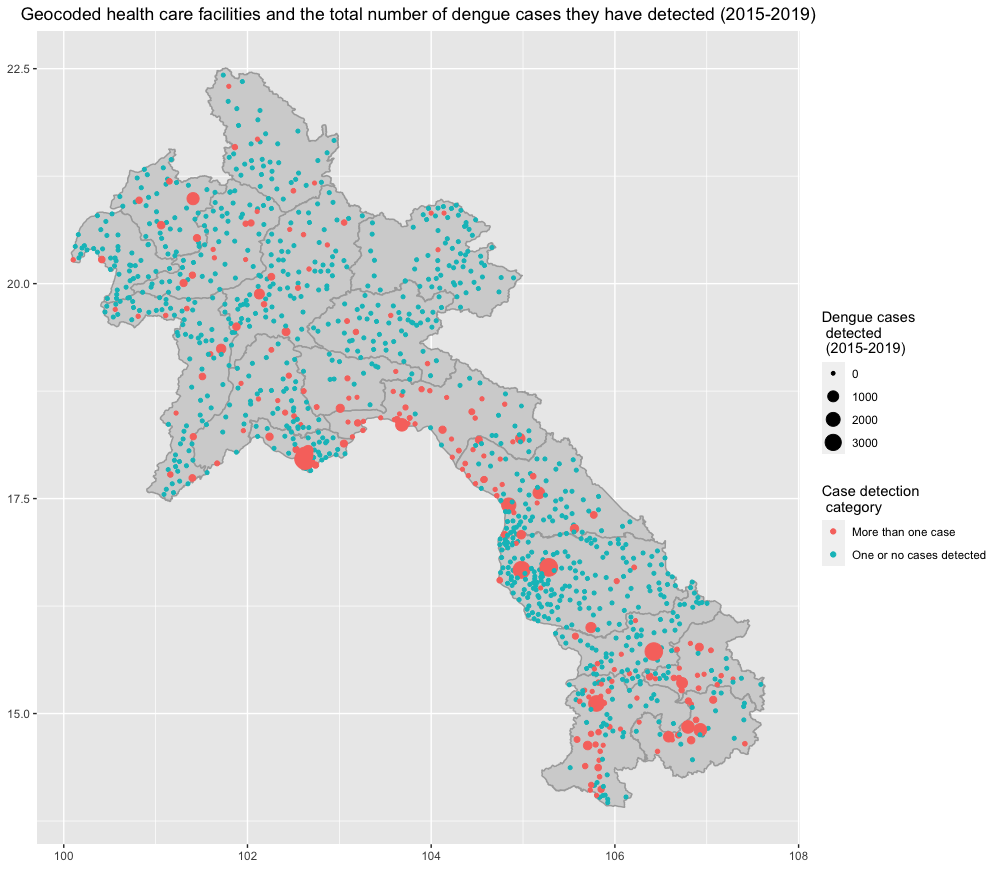


Figure S1. Locations of geocoded health care facilities (HCFs) (n = 1,142) and cumulative dengue cases detected at each facility over the study period. Geographical coordinates of 139 of the 1,281 HCFs were not available.

*
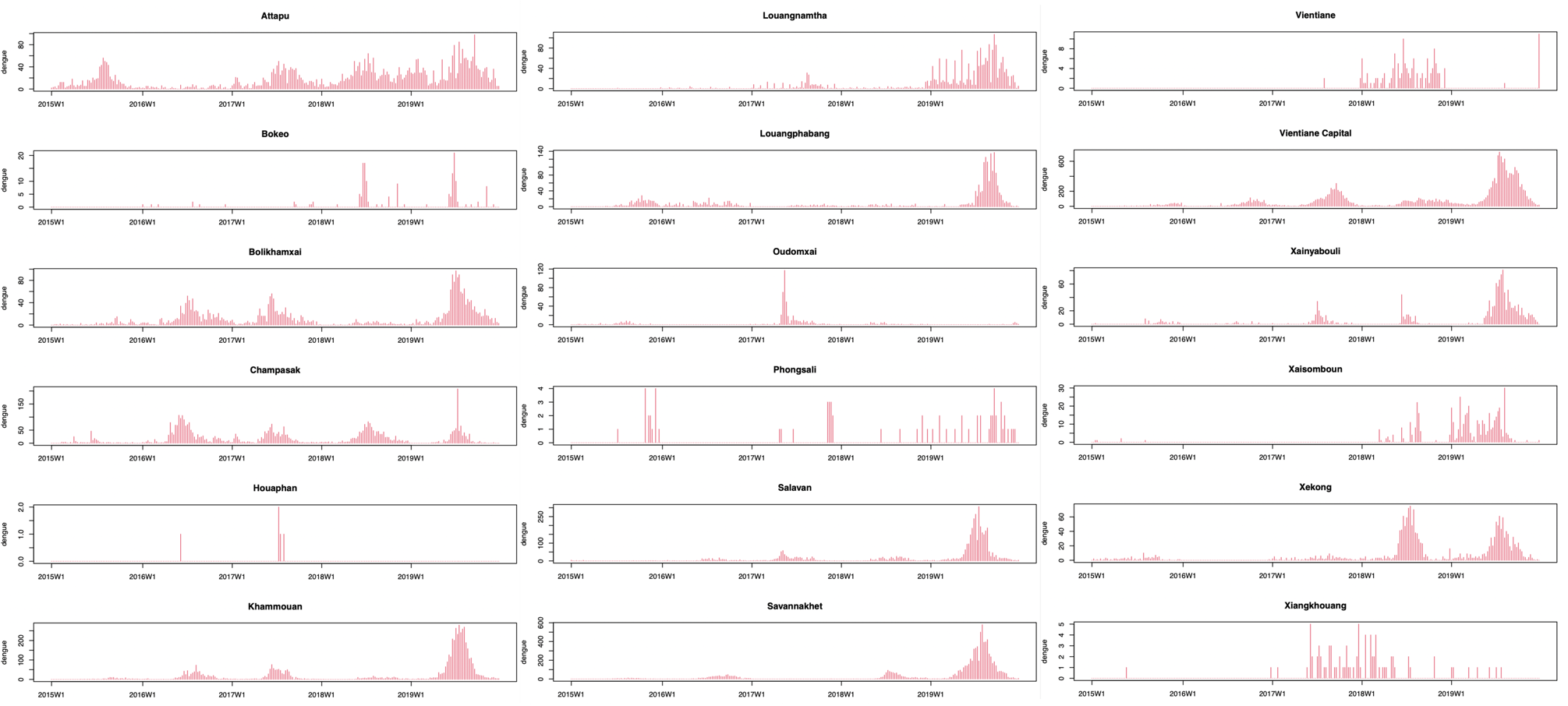
* Figure S2. Time-series plots of weekly dengue cases in 18 jurisdictions over the study period (2015-2019)
